# A Signaling Pathway at the Heart of Muscle Development

**DOI:** 10.1371/journal.pbio.0030358

**Published:** 2005-09-06

Unlike virtually every other type of cell, muscle cells contain dozens or even hundreds of nuclei. These multinucleate cells, called myofibers, form by fusion of precursor cells, called myoblasts, with “founder cells.” In the fruit fly, embryonic founder cells are formed by a well-known signaling pathway, but the same mechanism is not used to form adult founder cells. In this issue, K. VijayRaghavan and colleagues identify several key molecules involved in adult founder cell formation, and show that the process occurs through a novel mechanism.

In the fly embryo, founder cells differentiate from myoblasts through the actions of a membrane-bound receptor called Notch, an important player in several “signaling cascades” that use environmental signals to trigger changes in gene expression. However, in previous work, the authors have shown that Notch does not play a role in establishing adult founder cells. Instead, several clues pointed to the Fibroblast growth factor (FGF) family of receptors, one of which, in the fly, is called Heartless. Among other locations, Heartless is found on the surface of adult myoblasts in the abdomen. Reducing its expression in these cells, the authors showed, reduced the number of founder cells, while elevating it increased them.

But since Heartless is found in all adult myoblasts, it could not be responsible by itself for converting a myoblast into a founder cell. Another protein, called Heartbroken, seemed like a good candidate, since it functions exclusively within the FGF pathway. The authors showed that while the gene for Heartbroken is initially expressed in all myoblasts, over time its expression becomes restricted to those cells that develop into founder cells. Furthermore, by artificially maintaining Heartbroken expression, the authors dramatically elevated the number of founders in developing muscle, strengthening the case that Heartbroken is a key promoter of founder cell development.

But since both Heartless and Heartbroken are initially present in early myoblasts, what prevents wholesale Heartless signaling and premature, widespread founder cell formation? The authors show that a third factor, called Sprouty, declines in expression as founders are specified, and is not detected after founders are established. Sprouty is known to be a negative regulator of FGF signaling. VijayRaghavan and colleagues suggest that Sprouty interferes with Heartless signaling in early myoblasts, preventing founder cell formation even in the presence of Heartbroken. The gradual decline in the level of Sprouty may then “release the brakes” on Heartless signaling. Not every myoblast becomes a founder cell at that point, though, because the level of Heartbroken has also declined. Exactly which cells will maintain sufficient Heartbroken to become founders, and how those cells are specified, remains to be worked out.[Fig pbio-0030358-g001]

**Figure pbio-0030358-g001:**
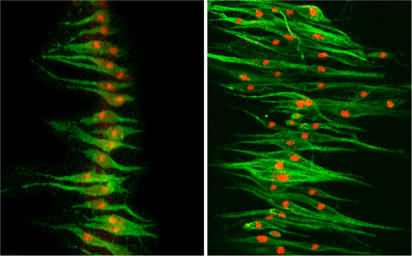
Drosophila adult muscle founder cells are selected by the Heartless signaling pathway. Overexpression of components of the Heartless pathway increases the number of founder cells (right) compared to normal expression (left)

Still more remains to be discovered about the development of muscle in the adult fly. The significance of this work lies in identifying the FGF pathway as a critical component in muscle cell development, which provides leads that can be used to fill in the missing members of the pathway. While the details of vertebrate muscle development differ, the FGF pathway is known to be involved there too, and this work may shed light on aspects of that process as well. —*Richard Robinson*

